# Supplementary material for: Perceptions of the seriousness of major public health problems during the COVID-19 pandemic in seven middle-income countries
Source: Commun Med (Lond). 2023 Dec 21;3:193. doi: 10.1038/s43856-023-00377-8 (PMC10739711; doi:10.1038/s43856-023-00377-8)
Supplement: Supplementary file 1 — Supplementary Information [file 43856_2023_377_MOESM1_ESM.pdf]

Supplementary Information

Supplementary Methods: Sample Selection, Execution, and Weighting

The survey was administered online in February and March of 2022 by YouGov using respondents selected from their country-specific opt-in panels.[1] To be eligible for inclusion, individuals had to be 18 or older and have regular access to a device connected to the internet at the time of the survey (e.g., cell phone, computer, or tablet). The fraction of those eligible for the survey varies across countries. Information from YouGov at the time the sampling weights were developed shows this fraction to be 62% in Colombia, 34% in India, 18% in Kenya, 28% in Nigeria, 56% in South Africa, 16% in Tanzania, and 50% in Vietnam. Later information sources suggest that there is now substantially higher internet penetration and that officially reported statistics for the African countries is considerably lower than that reported in one of the standard industry sources [1,2]. Characteristics of the internet-connected population were drawn from the following sources: random probability samples used in the Latin American Public Opinion Project 2019 (Colombia), the Pew Global Attitudes Survey 2017 (Vietnam), the Pew Global Attitudes Survey 2019 (India), and the Afrobarometer survey 2019 (Kenya, Nigeria, South Africa and Tanzania). The sample was weighted by YouGov on age and gender using a propensity score approach to this internet-connected sampling frame, not the general population. This sampling frame tends to be skewed toward both male and younger respondents.

Across countries, the weighted sample median age is 31.9; with this fraction falling in a narrow band from 29.4 in Nigeria to 33.5 in South Africa. Note that these are conditional on being

---

[1] For more on YouGov's treatment of online panel issues see its responses to the 28 questions Esomar.org [European Society for Opinion & Marketing Research] considers to be important considerations in assessing and using the results from such surveys: http://cdn.yougov.com/cumulus_uploads/document/t3r5k565j5/ESOMAR_28.pdf

18 or older. The weighted male fraction is 56.3. This fraction exhibits more variability. At the low end are Vietnam (49.5 male), Colombia (50.0) and South Africa (50. 9). In the middle, close to the overall sample median are Nigeria (56.4) and Kenya (56.6), while at the high end are Tanzania (62.4) and India (68.2). The demographic characteristics of the country-specific samples are relatively close to the characteristics of YouGov sampling frame for these two variables (i.e., age and gender), so the weighted and unweight results are quite similar.

Somewhat surprisingly, official country-level statistics as reported to international organizations like the United Nations or World Bank are not useful for making comparisons such as median age or the fraction who are female. The reason is that detailed age information is reported in age brackets and the lower end of relevant brackets is 15 and not 18 as in most surveys, including ours. This is because surveys interviewing those under 18 are treated very differently by the Institutional Review Board approval processes. The statistics we are interested in are those that are conditional on being 18 or older. Thus, YouGov and other survey organizations have to use results from rare large full probability-based samples using in-person interviews to obtain their baseline demographics. Academic access to these surveys is possible but they are not generally publicly available. For example, Pew's 2019 Global Values Survey in India has one of the lower internet access fractions and hence may be less representative of the general population than most of our middle-income countries. In Pew's 2019 Global Values Survey the median age in the raw data is 39.20, while in our survey the median age is 33.85. The percent female in the raw Pew data is 42.49 versus 31.83 in our sample of Indians with internet access. For income, the Pew survey uses 10 brackets. The average of these brackets for those with internet access is 5.9 while it is 4.5 for those without, with the largest divergence in the

lowest income bracket. In the Pew survey, 54.65% of those with internet access in India live in rural villages or on farms versus 69.18% of those without interact access.

The population eligible for inclusion in the survey, owing to its access to the internet, is also likely to be more educated, higher income, and more urban, although there is considerable variation in the sample on these characteristics. It is important to interpret results in this context.[2] They should produce unbiased estimates for this sampling frame but are not necessarily representative of a country's entire population. In particular, the seriousness of disease conditions that are more prevalent amongst the poor households and those living in rural areas may be less highly ranked by a sample of respondents taken from our internet-connected sampling frame. Depending on the country, the survey was administered in Afrikaans, English, Hindi, Spanish, Vietnamese and Zulu.

---

[2] Full-probability random samples of the general population using dwelling unit enumeration and in-person interviewing have become increasingly rare in academic research due to prohibitive cost and are now largely the domain of government statistical agencies. Survey researchers have turned to alternative less complete sampling frames from which they can draw random samples. Their properties have been changing over time. For instance, random digit dialing of phone landlines were capable of reaching over 90% of the U.S. population in the early 2000s, whereas that percentage is now under 30%. Thus, a U.S. phone survey that twenty years ago would have been (largely) representative of the country, would now interview largely older Americans who had not moved over that period. The growing penetration of internet access through computers and mobile phones has resulted in a shift in how survey research conducted across the world is affected by that mode of administration. As the penetration rate increases over time, surveys done using that mode become increasingly representative of a country's general population. Typically, there are not extremely large differences between those with internet access and those without, but that is not always the case and substantive divergences are common which calls for caution in assuming that the estimates based on a high-quality internet-based survey are representative of the entire population, even when it may be the best or only available estimate. A large effort is underway in the survey research community related to assembling/recruiting members for internet panels and how to sample from those panels. Major internet panel suppliers are quite transparent about the particular set of procedures they use (see Footnote 1 above for YouGov) in an effort to counter a proliferation of web-based surveys based on highly selected convenience samples which are not representative of any well-defined broad-based sampling frame.

Supplementary Methods: Elicitation Approach for Ranking Severity - Repeated Best-Worst Format

It is often difficult to get respondents to reliably rank order a long list of items. We use a best-worst (BW) elicitation to assist respondents in this task [3]. The BW elicitation format displayed the list of seven objects, the health problems (with their order of appearance randomized across respondents), and asked respondents to indicate the one they thought was the most serious and the one they thought was the least serious. Research has shown that it is easiest for respondents to report these two extremes. Then these two health problems are removed from the list. The remaining five health problems are then displayed, and the respondent is asked the same question, i.e., is instructed to check the most and least serious among these five. These two items are then removed, and the respondent is shown the set of the three remaining health problems and again asked for the most and least serious among this set. A respondent's answers to these three tasks provides their unique ranking of the seven health problems .

Obtaining a ranking order of more than seven items can be accomplished by deploying an experiment design that assigns individual respondents to subsets of the items. Such experimental designs (e.g., Youden) typically ensure that these subsets are balanced in the sense that across subsets each item appears an equal number of times, and, within a subset, each item appears the same number of times with each of the other items [4]. This would allow a complete ranking for the sample as a whole to be obtained for a much longer list of health problems. However, this would be at the expense of having only a partial rather than complete ordering for individual respondents.

A rank-ordered response variable is an improvement over the use of a Likert Scale because it avoids two problems. First, when respondents are presented with many questions and asked to record their answer for each on a Likert Scale, they often select the same response category on a Likert Scale for many questions. Second, respondents may use the same Likert response scale differently, with such differences being particularly pronounced in a cross-country context [5]. Also, it is simpler for a respondent to construct a complete ranking of a list of objects using a repeated best-worst format compared to a single question asking for a respondent's complete ordering.

The response data from the first BW question represent partially ranked data and are often analyzed using a set of techniques known as best-worst scaling that are particularly easy to calculate and communicate. These techniques simply compare differences in the counts of most and least serious or equivalently the percentage difference (the necessary representation when sample weights are used) of respondents choosing each health problem as the most serious and least serious. The unweighted counts and the weighted BW format percentage differences are displayed in Supplementary Table 1 by country and health problem as well as the row ranking of health problems for each country.

Other popular monotonic transformations like the ln(sqrt(Best/Worst)) typically produce the same rank ordering. Most strikingly, in the first round of Supplementary Table 1's most and least serious health problems, there is an extreme aggregate response in Vietnam where over 40% of the sample pick COVID-19 as their most serious health problem and a strong emphasis on drug and alcohol problems in the four African countries, where this drug and alcohol is ranked the most serious health problem in South Africa. Perhaps less obvious is that there is a striking degree

of heterogeneity. Even the least serious health problem in a country generally has about 5% of the sample ranking it as the most serious problem.

When a BW format question is repeated, a more complete ranking is obtained. While it is possible to display the percent of sample respondents that provide each ranking, there are 5040 permutations of a set of size 7, so some method of obtaining a (weighted) average rank ordering across respondents must be used. We turn to that issue next.

Supplementary Methods: Rank-Ordered Logit Model

There are many ways to analyze rank-ordered data ranging from a simple average of ranks to variations on rank choice voting. Most easily explainable and defensible procedures result in reasonably similar outcomes. We use here one of the most common approaches, the rank-ordered logit because of its wide-spread implementation and well-known statistical properties [6]. The rank-ordered logit model can be seen as a generalization of the conditional/multinomial logit model that is based on a complete (or partial) ranking rather than just a first choice alternative. Standard expositions can be found in many of the leading statistical packages. We used Stata 17.

Complications can arise when the assumption that the implicit scale factor across the ranks is constant is violated, as the ranking of middle-ranked alternatives is often statistically noisier. That is of little import here as both the naïve estimate obtained from simply averaging the ranks across respondents in a country and a more complicated variant of the rank-ordered logit model that allows the variance of the scale parameter to vary with the ranking level produce qualitatively similar results. This is not surprising since our model with country-specific

parameters on each health problem is essentially equivalent to estimating "market shares" in individual retail stores, a problem which is known to be robust to a wide range of heterogentity violations in the underlying individual data [7]. Detailed modeling of various scale parameters in a mixed or generalized multinomial logit sense [8, 9] might be warranted if the objective were to produce a precise estimate of a statistic like the average elasticity of the COVID ranking with respect to age, rather than simply noting that changes in age have little impact on the average rankings reported by our respondents. These approaches to allowing random parameters can be applied by recognizing that a rank-ordered response variable can be "exploded" into a group of choice sets that can be analyzed conditional logit type models for panel data [10].

Supplementary Methods: Rank-Ordered Logit Model Results

The two rank-ordered logit models used to produce the results in Figures 1, 2 and 3 are provided here. Figures 1 and 2 use only country indicators as covariates with the particular formulation used allowing each country to have its own coefficient on each health problem.[3] Figure 3 is based on a revised model that adds a set of eight covariates, four binary indicators, and four continuous variables to the first model. This allows us to assess how the common component of respondent characteristics (such as gender across countries) influence health problem ranking across countries. Exponentiating these coefficients is used to convert them to relative risk terms.

---

[3] A rank-ordered logit model using a single set of alternative specific constants (ASCs) for the seven health problems rather than the country specific version of those ASCs is clearly rejected using a likelihood ratio test at the p > .001 level [2*(-71645.39 - -72241.77)=1192.76, has a chi-square (df=42) distribution under the null that our respondent's rank ordered health data is adequately characterized by a single common set of ASC's. That single set of ASCs is: .7297 (respiratory), .4056 (COVID-19), .3565 (alcohol/drugs), HIV/AIDS (.3399), TB (.1263), malaria (-.2383), -.2383 (water) [all significant at the p < .001 level] provide a rank ordering for the sample as a whole.

These rank-ordered logit models tend to replicate the ordering of the different health problems “within” each country. Figures 2 and 3 are based on the rank ordering of the same health problem “across” countries. They use the continuous predictive scores derived from the rank ordered logit coefficients which provide a measure of the intensity of perceived seriousness on a common metric across countries. To see this, consider two countries where respondents rank a particular health problem as having the highest severity. In one country, the other health problems have low scores that are all close together, indicating that respondents see the severity of the other health problems as reasonably similar. In the other country, two other health problems have high scores close to the top rank health problem and the other four low scores are close together. The score of the second-ranked health problem in the first country will be lower than that of the third-ranked problem in the second country.

All model coefficients for the rank-ordered logit model are presented in an Excel file in Supplementary Data 2.

Supplementary Methods: Initial Best-Worst Question

The following is the first of a sequence of three questions that respondents saw that elicit responses using a best-worst format , here cast in terms of "Most Serious" and "Least Serious" among items in the list presented:

Which of these health issues do you think will be the most and least serious problems in your community over the next five years:

Check ONE circle indicating the item you think is the most serious and check ONE circle for the item that you think is the least serious.

	MOST SERIOUS	LEAST SERIOUS
Malaria	○	○
Lung cancer and respiratory diseases caused by air pollution and smoking	○	○
Tuberculosis	○	○
COVID-19	○	○
HIV/AIDS	○	○
Water borne diseases like diarrhea	○	○
Alcoholism and drug use	○	○

In interpreting the results of this severity assessment task, it is important to understand the specific context in which these choice tasks are set. These best-worst questions asked respondents about the "seriousness of health problems in their community over the next five years." Respondents' answers should be affected by all four of the descriptors (seriousness, health, community, and five years). "Seriousness" is a subjective interpretation for the respondent and is distinct from any particular medical outcome like the number of deaths. Understandably, however, one would expect that the number of deaths due to a disease would influence perceptions of seriousness. The term "health problem" is used to encompass "alcohol and drugs" as a problem to be ranked. "Community" denotes the local area as perceived by the respondent rather than the country as a whole. "Five years" is an intermediate time horizon, not the distant future. We intentionally did not include cancer, heart disease, and other chronic

conditions because, although these are among the leading causes of death in all countries, they are most often diseases of the elderly, and therefore the least likely to substantively respond to public health interventions over the indicated five-year time horizon.

Supplementary Methods: Questions Underlying the COVID-19 Knowledge Scale

We calculated our COVID-19 knowledge index by assigning a "+1" to each correct response, a "0" to a don't know response, and a "-1" to an incorrect response and summing the answers to all the questions.

Indicate whether you agree, disagree or are not sure about each of these statements:

	Disagree	Agree	Not Sure
Young adults are immune and cannot be infected with COVID-19.	○	○	○
Standing 2 meters apart helps reduce COVID-19's spread.	○	○	○
Someone is less likely to get COVID-19 outdoors than doing the same activity indoors.	○	○	○
You can have COVID-19 and infect others without showing any symptoms.	○	○	○
Someone getting COVID-19 is no more likely to die than if they get influenza.	○	○	○

Indicate whether you agree, disagree or are not sure about each of these statements:

	Disagree	Agree	Not Sure
Face masks do not help reduce COVID-19's spread.	○	○	○
Most scientists and doctors believe that the new Covid-19 vaccines are safe and effective.	○	○	○
The first people infected with COVID-19 were in China.	○	○	○
Avoiding crowds reduces your chances of infection with COVID-19.	○	○	○
Scientists believe that the COVID-19 virus has stopped mutating, and we are unlikely to face any new variants in the future.	○	○	○

Supplementary References

[1] World Bank. World Telecommunication/International Telecommunication Union Indicators Database. Accessed on 6th November, 2022.

[2] Kimp, S. Digital 2022: Local Country Headlines Report, January 2022.

https://datareportal.com/reports/digital-2022-local-country-headlines

[3] Louviere, J.J., Flynn, T.N. & Marley, A.A.J. *Best-worst scaling: Theory, Methods and Applications* (Cambridge University Press, 2015).

[4] Raghavarao, D. *Constructions and combinatorial problems in design of experiments* (New York: Dover, 1988).

[5] Heine, S. J., et al. What's wrong with cross-cultural comparisons of subjective Likert scales? The reference-group effect. *Journal of Personality and Social Psychology* **82**, 903–918 (2002).

[6] Greene, W.H. *Econometric analysis*. 8th ed. (New York: Pearson, 2017).

[7] Allenby, G.M. & Rossi, P.E. There is no aggregation bias: Why macro logit models work. *Journal of Business and Economic Statistics* **9**, 1-14 (1991).

[8] Train, K.E. *Discrete choice methods with simulation, 2nd ed.* (New York: Cambridge University Press, 2009).

[9] Fiebig, D.G., et al. The generalized multinomial logit model: accounting for scale and coefficient heterogeneity. *Marketing Science* 29, 393-421 (2010).

[10] Chapman, R. G., & Staelin, R. Exploiting rank ordered choice set data within the stochastic utility model. *Journal of Marketing Research*, **19**, 288-301 (1982).

Supplementary Table 1: Best Worst Scaling Estimates Based on First Question Response*

	Alcohol & Drugs	COVID	HIV/AIDS	Malaria	Resp. Disease	Tuberculosis	Water-Borne
Colombia	12.3/(5)<br>196/150	34.2/(6)<br>280/142	8.3/(4)<br>121/90	-65.9/(1)<br>31/149	80.1/(7)<br>397/45	-44.6/(3)<br>44/115	-56.7/(2)<br>77/285
India	-13.3/(4)<br>137/160	46.5/(6)<br>417/42	-6.0/(5)<br>139/150	-56.3/(3)<br>189/46	68.3/(7)<br>326/62	-60.5/(2)<br>27/87	-68.0/(1)<br>48/268
Kenya	47.9/(6)<br>350/123	7.0/(5)<br>240/211	1.1/(4)<br>144/134	-32.8/(3)<br>99/200	50.7/(7)<br>258/82	-52.9/(2)<br>38/125	-66.4/(1)<br>49/254
Nigeria	36.5/(6)<br>265/117	-2.7/(4)<br>211/224	-24.2/(3)<br>96/157	24.94/(5)<br>298/173	36.6/(7)<br>198/95	-63.0/(1)<br>29/135	-43.4/(2)<br>67/172
S. Africa	55.9/(7)<br>318/110	14.8/(5)<br>264/197	-0.7(4)<br>151/152	-79.8/(1)<br>34/296	49.7/(6)<br>218/72	-12.3/(3)<br>63/80	-51.8/(2)<br>62/202
Tanzania	44.7/(6)<br>248/89	5.1/(4)<br>231/199	19.3/(5)<br>165/123	-32.3/(3)<br>112/218	56.5/(7)<br>248/80	-33.3/(2)<br>49/97	-45.1/(1)<br>80/195
Vietnam	0.1/(4)<br>116/122	72.9/(7)<br>503/74	22.6/(5)<br>145/96	-76.6/(2)<br>29/217	70.5/(6)<br>285/50	-39.5/(3)<br>46/108	-80.8/(1)<br>43/377

*First row: most – least serious weighted percentage/(row rank); Second row: unweighted most serious count/least serious count.
