# Supplementary material for: Perceptions of the seriousness of major public health problems during the COVID-19 pandemic in seven middle-income countries
Source: Commun Med (Lond). 2023 Dec 21;3:193. doi: 10.1038/s43856-023-00377-8 (PMC10739711; doi:10.1038/s43856-023-00377-8)
Supplement: Supplementary file 4 — Description of Additional Supplementary Files [file 43856_2023_377_MOESM4_ESM.pdf]

Description of Additional Supplementary Files

File Name: Supplementary Data 1
Description: Data for Figure 1

File Name: Supplementary Data 2
Description: Model coefficients for the rank-ordered logit model
