# Supplementary material for: Perceptions of the seriousness of major public health problems during the COVID-19 pandemic in seven middle-income countries
Source: Commun Med (Lond). 2023 Dec 21;3:193. doi: 10.1038/s43856-023-00377-8 (PMC10739711; doi:10.1038/s43856-023-00377-8)
Supplement: Supplementary file 5 — Reporting Summary [file 43856_2023_377_MOESM5_ESM.pdf]

nature portfolio

Corresponding author(s): Dale Whittington

Last updated by author(s): 9/23/2023

Reporting Summary

Nature Portfolio wishes to improve the reproducibility of the work that we publish. This form provides structure for consistency and transparency in reporting. For further information on Nature Portfolio policies, see our Editorial Policies and the Editorial Policy Checklist.

Statistics

For all statistical analyses, confirm that the following items are present in the figure legend, table legend, main text, or Methods section.

n/a	Confirmed	
☐	☒	The exact sample size (*n*) for each experimental group/condition, given as a discrete number and unit of measurement
☐	☒	A statement on whether measurements were taken from distinct samples or whether the same sample was measured repeatedly
☐	☒	The statistical test(s) used AND whether they are one- or two-sided<br>*Only common tests should be described solely by name; describe more complex techniques in the Methods section.*
☐	☒	A description of all covariates tested
☐	☒	A description of any assumptions or corrections, such as tests of normality and adjustment for multiple comparisons
☐	☒	A full description of the statistical parameters including central tendency (e.g. means) or other basic estimates (e.g. regression coefficient) AND variation (e.g. standard deviation) or associated estimates of uncertainty (e.g. confidence intervals)
☐	☒	For null hypothesis testing, the test statistic (e.g. *F*, *t*, *r*) with confidence intervals, effect sizes, degrees of freedom and *P* value noted<br>*Give P values as exact values whenever suitable.*
☒	☐	For Bayesian analysis, information on the choice of priors and Markov chain Monte Carlo settings
☒	☐	For hierarchical and complex designs, identification of the appropriate level for tests and full reporting of outcomes
☐	☒	Estimates of effect sizes (e.g. Cohen's *d*, Pearson's *r*), indicating how they were calculated

Our web collection on statistics for biologists contains articles on many of the points above.

Software and code

Policy information about availability of computer code

Data collection	Survey data was collected by YouGov.com for this project using respondents from their country specific panels and administered via computers, mobile phones and tablets.
Data analysis	Data analysis was performed using Stata 17, a standard statistical package.

For manuscripts utilizing custom algorithms or software that are central to the research but not yet described in published literature, software must be made available to editors and reviewers. We strongly encourage code deposition in a community repository (e.g. GitHub). See the Nature Portfolio guidelines for submitting code & software for further information.

Data

Policy information about availability of data

All manuscripts must include a data availability statement. This statement should provide the following information, where applicable:

- Accession codes, unique identifiers, or web links for publicly available datasets
- A description of any restrictions on data availability
- For clinical datasets or third party data, please ensure that the statement adheres to our policy

Data and computer code used in this paper will be posted for public access without restriction on the Environment for Development website upon publication.

Human research participants

Policy information about studies involving human research participants and Sex and Gender in Research.

Reporting on sex and gender	The study reports on one odds ratio for "female" based on an indicator variable for that response category from one of YouGov's standard demographic question.
Population characteristics	Sampling frame used was the Internet connected population, age 18 and above, in Colombia, India, Kenya, Nigeria, South Africa, Tanzania, and Vietnam.
Recruitment	Participants were recruited by YouGov from their country specific panels. The obvious selection criteria here is having a connection to the Internet.
Ethics oversight	The survey received Institutional Review Board (IRB) approval from the Swedish Ethical Review Authority, Research Ethics Board at the University of Alberta, and Research Review Board at Wageningen University in the Netherlands. Participants in the YouGov internet panel gave informed consent.

Note that full information on the approval of the study protocol must also be provided in the manuscript.

Field-specific reporting

Please select the one below that is the best fit for your research. If you are not sure, read the appropriate sections before making your selection.

☐ Life sciences ☒ Behavioural & social sciences ☐ Ecological, evolutionary & environmental sciences

For a reference copy of the document with all sections, see nature.com/documents/nr-reporting-summary-flat.pdf

Behavioural & social sciences study design

All studies must disclose on these points even when the disclosure is negative.

Study description	Study is a quantitative analysis of cross-sectional survey data from seven large developing countries.
Research sample	Sample selected from the Internet enable population in Colombia, India, Kenya, Nigeria, South Africa,Tanzania, and Vietnam chosen from YouGov.com's country specific panels. Paper discuss at length the difference between this sampling frame and a representative sample of each of the country's population 18 and over.
Sampling strategy	Random sample drawn to be representative of each of the country's Internet enable population, determine by large large full probability reference surveys. No explicit sample size calculation was made before hand, but sample size of 1200 in each country (8400 respondents overall) is substantial larger than most commercial public opinion surveys.
Data collection	Survey was administered on computers, mobile phones, and tablets by YouGov.com who upon completion and after quality control checks supplied the researchers with a completed dataset.
Timing	Data collected January through March 2022, with most observations collected in February.
Data exclusions	None
Non-participation	None
Randomization	Randomization not used in this paper.

Reporting for specific materials, systems and methods

We require information from authors about some types of materials, experimental systems and methods used in many studies. Here, indicate whether each material, system or method listed is relevant to your study. If you are not sure if a list item applies to your research, read the appropriate section before selecting a response.

Materials & experimental systems

n/a	Involved in the study
☒	☐ Antibodies
☒	☐ Eukaryotic cell lines
☒	☐ Palaeontology and archaeology
☒	☐ Animals and other organisms
☒	☐ Clinical data
☒	☐ Dual use research of concern

Methods

n/a	Involved in the study
☒	☐ ChIP-seq
☒	☐ Flow cytometry
☒	☐ MRI-based neuroimaging
